# Comparison of Lyophilized Glutaraldehyde-Preserved Bovine Pericardium with Different Vascular Prostheses for Use as Vocal Cords Implants: Experimental Study

**DOI:** 10.1155/2015/351862

**Published:** 2015-05-13

**Authors:** J. Raúl Olmos-Zuñiga, Rogelio Jasso-Victoria, Miguel Gaxiola-Gaxiola, Avelina Sotres-Vega, Claudia Hernández-Jiménez, Matilde Baltazares-Lipp, Fernando Arredondo del Bosque, Patricio Santillan-Doherty

**Affiliations:** ^1^Department of Surgical Research, Instituto Nacional de Enfermedades Respiratorias Ismael Cosio Villegas, Tlalpan, 14080 Mexico City, DF, Mexico; ^2^Department of Morphology, Instituto Nacional de Enfermedades Respiratorias Ismael Cosio Villegas, Tlalpan, 14080 Mexico City, DF, Mexico; ^3^Department of Otolaryngology-Head and Neck Surgery, Instituto Nacional de Enfermedades Respiratorias Ismael Cosio Villegas, Tlalpan, 14080 Mexico City, DF, Mexico; ^4^Medical Direction, Instituto Nacional de Enfermedades Respiratorias Ismael Cosio Villegas, Tlalpan, 14080 Mexico City, DF, Mexico

## Abstract

This study compared the use of lyophilized glutaraldehyde-preserved bovine pericardium (LGPBP), polytetrafluoroethylene (PTFE), polyethylene terephthalate (PET), and Teflon felt (TF) as implants for vocal cords (VC) medialization and aimed to assess the endoscopic, macroscopic, and microscopic VC changes after medialization in a canine model. In 18 mongrel dogs, the right VC were medialized with LGPBP and the left were implanted as follows: Group I (*n* = 6): LGPBP and PTFE; Group II (*n* = 6): LGPBP and PET; Group III (*n* = 6): LGPBP and TF. Surgical handling of the implants was compared. Three months after surgery, macroscopic and microscopic changes of VC and implants were evaluated. LGPBP offered the best surgical handling (*p* = 0.005, Kruskal-Wallis). TF implants showed extrusion (*p* = 0.005, Kruskal-Wallis) and severe inflammation. All VC formed fibrous capsules around the implants; the ones developed by LGPBP implants were thinner (*p* = 0.001, ANOVA, Tukey). VC implanted with synthetic materials showed eosinophilic infiltration (*p* = 0.01, Kruskal-Wallis). We concluded that the LGPBP could be used as an implant for VC medialization because it is biocompatible, easy to handle and remove during surgical procedures, and nonabsorbable or extrudable and produces an inflammatory reaction similar to PTFE and PET.

## 1. Introduction

Medialization of paralyzed vocal cords (VC) by type I thyroplasty is a transcervical surgical procedure in which a prosthesis is implanted in the entire length of the vocal fold through a window made in the thyroid cartilage and placed into a small dissection sac within the paraglottic space to transfer the VC to the midline to improve glottic closure [1–3].

Polytetrafluoroethylene (PTFE) (Goretex), polyethylene terephthalate (PET) (Dacron), and Teflon felt (TF) are materials that have been used for the construction of vascular prostheses and meshes to treat abdominal hernias. These materials are biocompatible and allow graft coverage with tissue that is similar to normal [4]. Experimental and clinical studies have reported that PTFE is an effective material for VC medialization, given that it is biocompatible; produces minimal inflammation and foreign body reaction; has a low rate of extrusion, migration and infection; and may be easily increased, adjusted, or removed during revision surgery [5–8]. Additionally, PET and TF are materials with similar characteristics to PTFE, but there are no reports in the literature of their use for VC medialization. However, the cost of these materials in Mexico is very high and patients requiring treatment generally have poor healthcare coverage and restricted access to these implants, so it is necessary to seek other materials with these characteristics.

Glutaraldehyde-preserved bovine pericardium (GPBP) is a material that was initially used to prepare heart patches, valves, and vessels. We have obtained good results using a 0.5% glutaraldehyde solution for preservation and lyophilization (performed to reduce glutaraldehyde residue, facilitating its storage and transport) for the reconstruction of thoracoabdominal wall defects [9], nonanatomical lung resection [10, 11], surgical repair of nasal septal perforations [12], and closure of atrial septal defects [13]. In these studies, it has been found that these materials are biocompatible, safe and the cost of its preparation is much less than the cost of synthetic materials [9–13], which is why their use as VC implants may be a good alternative in Mexico. The aim of this study was to compare the use of LGPBP, PTFE, PET, and TF as implants for VC medialization and to assess the endoscopic, macroscopic, and microscopic VC changes after medialization in a canine model.

## 2. Materials and Methods

### 2.1. Preparation of LGPBP

The pericardium was obtained from bovines between 6 and 18 months old at the local abattoir after euthanasia with a penetrating captive-bolt pistol. Local mechanical cleansing was performed manually by dissecting off excess pericardial fat. The pericardium was submerged in 4°C saline solution in order to be transported. Further cleansing was performed in the laboratory to remove all fatty and connective tissue by dissecting it off the pericardium with clean dry gauze; clean pericardium was further washed with 4°C Hank's solution (SIGMA Chemical Co., St. Louis, MO) in a container with an electromagnetic agitator for 6 h. The pericardium was then mounted on 15 cm diameter plastic frames and submerged in 0.5% glutaraldehyde in 0.1 M phosphate-buffered saline (pH 7.4) at 4°C for at least 15 days before testing. After the initial preservation period, samples were taken from both the preservation solution and the pericardium for microbiological cultures [9–13].

After the GPBP was prepared, it was cut into strips 3 cm wide and 10 cm long. These strips were washed for 1 h in saline solution (0.9% Sodium Chloride Solution, Pisa, Guadalajara, Mexico) to remove excess glutaraldehyde. They were then placed in sterile crystal containers and frozen for one hour at −70°C. Subsequently, they were lyophilized with a 10 mbar vacuum at a temperature of −55°C for a period of 4 h. After lyophilization, each sample of LGPBP was packed, sterilized with gas (Sterrad, low-temperature hydrogen peroxide gas plasma sterilizing process, Johnson and Johnson Medical, Inc., New Brunswick, NJ, USA), and stored at room temperature before surgical application. Small squares of 5 mm × 5 mm were taken for microbiological culture just before implantation in the VC [11–13].

### 2.2. Experimental Animals

The study was conducted using 18 healthy mongrel dogs regardless of sex or age, weighing between 15 and 20 kg. This protocol was reviewed and approved by the Bioethics Committee of the Instituto Nacional de Enfermedades Respiratorias Ismael Cosío Villegas (INERICV) and was carried out according to the technical specifications for the care and use of laboratory animals of the Official Mexican Norm [14] and the Guide for the Care and Use of Laboratory Animals prepared by the National Institutes of Health of the USA [15].

The animals were divided into 3 study groups, and all underwent thyroplasty and medialization of both VC as follows.

*Group I *(*n* = 6). Medialization of the right VC with LGPBP and the left with PTFE (Gore acuseal patch, W.L. Gore and Associates, Ariz, USA).

*Group II *(*n* = 6). Medialization of the right VC with LGPBP and the left with PET (Dacron patch Hemashield Gold, Boston Scientific, Wayne, NJ, USA).

*Group III *(*n* = 6). Medialization of the right VC with LGPBP and the left with TF strips (Teflon patch, Boston Scientific Medi-tech, NJ, USA).

### 2.3. Anesthesia and Surgical Technique

The animals were anesthetized with xylazine hydrochloride (0.1 mg/kg) (Rompun, Bayer, Leverkusen, Germany) and propofol (6 mg/kg, IV) (Recofol, PISA, Guadalajara, Mexico) intravenously [16]. During surgery, supplemental intravenous doses of propofol were administered as needed to maintain adequate anesthesia.

Before surgery, LGPBP implants were rehydrated for 30 min in saline solution [11, 12]. Immediately, the biological and synthetic implants were cut into strips 3 mm wide by 5 cm long.

A cervical horizontal incision was made in the skin over the midline of the thyroid cartilage. By dissecting the subcutaneous tissue and muscle planes, the thyroid cartilage was exposed, and a microfenestra 4 mm wide by 4 mm long was created with a number 11 scalpel blade at the level of the VC. Subsequently, the inner perichondrium was dissected and the implants were folded over themselves to be introduced through the microfenestra within the subperichondrial space until the required volume was achieved. Special care was taken not to medialize the VC excessively so as not to reduce the glottic space and cause breathing problems. Finally, the implants were fixed behind the thyroid lamina, and the microfenestra was closed by external suture of the perichondrium and the process was concluded by conventional closure. Placement of the implant was visualised endoscopically during surgery. Medialization of the VC was considered correct when they were observed in their normal anatomical position at rest. This approach was followed because, in animals, it is not possible to determine the size of the implant material according to voice quality.

All the animals were treated preoperatively and for 5 days after, with enrofloxacin (5 mg/kg) (Baytril, Bayer, Leverkusen, Germany) intramuscularly (im) and flunixin meglumine (0.1 mg/kg) (im) (Napzin, Pisa Agropecuaria S.A. de C.V. Tula, Hidalgo, Mexico) as analgesic [16].

All surgical procedures were performed by the same surgical team.

### 2.4. Evaluation

#### 2.4.1. Surgical Handling of the Materials

The surgical handling of the materials was assessed according to their flexibility, ease of cutting and shaping (giving them the correct size and shape), and ease of insertion or removal from the VC during surgery and at the end of the study. This assessment was conducted using the following scale: grade 1 (easy to handle): easy to fold, cut, and give shape and offers no resistance to being inserted or removed from the microfenestra; grade 2 (intermediate handling): easy to fold without resistance to being inserted or removed from the microfenestra, but difficult to cut and give shape; and grade 3 (difficult handling): difficult to fold, cut, give shape, and insert or remove from the microfenestra.

#### 2.4.2. Clinical Evaluation

The animals were clinically evaluated before surgery, daily during the first postoperative week, and weekly throughout the rest of the study. In this evaluation, the presence of dysphonia, hoarseness, cough, stridor, or dyspnea was assessed using a scale based on the severity of the clinical changes: grade 1 (absent); grade 2 (mild); grade 3 (moderate); and grade 4 (severe).

#### 2.4.3. Endoscopic Evaluation

An endoscopic evaluation was performed before and after medialization and each week thereafter. In this endoscopic evaluation, the motility and position of both VC were assessed with respect to the glottic space, and we noted the presence of a reduced glottic space, inflammation, granulomas, infection, and migration or extrusion of the implant.

#### 2.4.4. Macroscopic Evaluation

Three months after surgery, all animals were euthanized with an overdose of sodium pentobarbital (80 mg/kg, IV) (Anestesal, Pfizer, State of Mexico, Mexico). The larynges were obtained, and the following were macroscopically evaluated: the position of the VC, any absorption, migration or extrusion of the implant, granuloma formation, infection, the presence of a fibrous capsule, and tissue growth into the implant and between its layers.

#### 2.4.5. Microscopic Evaluation

The larynges were fixed in 10% formaldehyde. Subsequently, serial axial slices were created and embedded in paraffin, stained with hematoxylin-eosin and Masson's trichrome, and studied histologically to examine the formation of a fibrous capsule and its thickness; the degree of inflammation; the presence of calcifications; the presence of inflammatory cells, such as foreign body giant cells, eosinophils, neutrophils, or lymphocytes; and the neoformation of vessels. The evaluation was conducted using a semiquantitative scale in which each evaluated parameter received a percentage score according to the severity of histological changes (0–10% absent, 11–25% mild, 26–50% moderate, and 51–100% severe). The microscopic evaluation and measurement of the thickness of the fibrous capsule were performed blindly by two pathologists who studied the slides twice.

### 2.5. Statistical Analysis

All data were analyzed using SPSS 18.0 for Windows (SPSS Inc., Chicago, IL, USA) and the numerical data were expressed as mean ± standard error (EE). To compare the nonparametric findings for the three materials, Kruskal-Wallis tests were used. To compare parametric findings, analysis of variance (ANOVA) and Tukey's test were used; *p* values < 0.05 were considered significant.

## 3. Results

All microbiological cultures performed on LGPBP implants were negative.

### 3.1. Findings of Surgical Handling of the Materials

According to the surgical team, only Teflon implants were difficult to cut and give the appropriate size and shape for insertion into the VC. LGPBP implants showed greater flexibility and less memory (*p* = 0.005, Kruskal-Wallis) than synthetic materials, which had a tendency to curl at the edges. TF strips had to be inserted into the microfenestra with a dissecting forceps. LGPBP, PTFE, and PET were easier to remove than TF (*p* = 0.005, Kruskal-Wallis) (Table 1).

### 3.2. Clinical Findings

All animals survived the surgical procedure. Three of the animals (50%) who were implanted with TF (*p* < 0.01, ANOVA, Tukey) did not complete the study because they were removed from the investigation due to having cough, stridor, and halitosis from the fifth week after surgery, which is why the implant had to be removed 6 weeks after surgery in 2 cases and 8 weeks after surgery in 1 case. None of the animals who underwent medialization with LGPBP, PTFE, and PET and the remaining 3 animals with TF showed differences in clinical evaluation.

### 3.3. Endoscopic Findings

**Figure 1:**
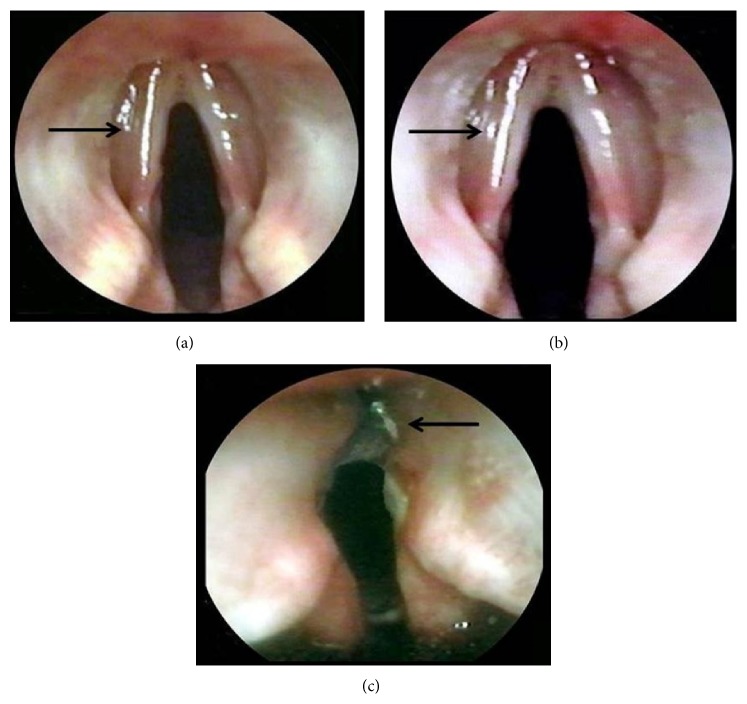
Final endoscopy of the VC medialized. Showing the position of VC after medialization without granulomas, extrusion of the implant, or narrowing of the glottic space in animals treated with (a) LGPBP (Arrow) and PTFE, (b) LGPBP (Arrow), and PET. (c) Thickened of VC and extrusion of TF (Arrow).

Endoscopically, all animals showed VC motility during all study time. The animals who underwent medialization with LGPBP, PTFE, and PET maintained the same volume after medialization, and the position of both VC with respect to the glottis remained unchanged. All animals developed inflammation and edema of the VC during the first postoperative week, which was mild in those implanted with LGPBP (*p* = 0.005, Kruskal-Wallis) and moderate in those with PTFE and PET; however, inflammation disappeared in the second postoperative week. At the end of the study, none of the animals implanted with LGPBP, PTFE, or PET presented glottic space narrowing, granulomas, or extrusion of the implant, but those implanted with synthetic materials were thicker than those with pericardium (Table 2) (Figures 1(a) and 1(b)). In the TF group, inflammation and edema were severe with respect to the other three materials (*p* = 0.001, Kruskal-Wallis); these findings in the 3 animals that completed the study disappeared 4 weeks after surgery, while the other 3 dogs in this group had infection and extrusion of the implant (*p* = 0.005, Kruskal-Wallis test), 6 weeks after surgery in 2 animals and 8 weeks after surgery in 1 (16.7%) (Figure 1(c)). At the end of the study, the VC implanted with TF had thickened more than those with other materials, and in 1 case, an ulcer appeared in the VC (Table 2).

### 3.4. Macroscopic Findings

Macroscopic study of the VC medialized with LGPBP, PTFE, and PET did not show swelling, absorption, or extrusion of the implant or tissue growth within the implant or its layers. The VC medialized with TF in the dogs that reached the end of the study showed severe inflammation; in the other 3 cases, the presence of infection and extrusion of the implant were confirmed. In all axial slices from the animals that completed the study, the formation of a fibrous capsule around the implant was observed, which was measured in millimeters during the histological study.

### 3.5. Microscopic Findings

**Figure 2:**
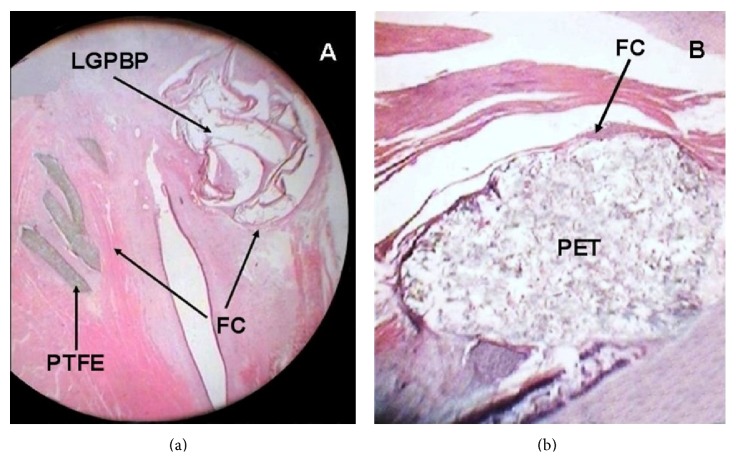
Microscopic findings. Showing fibrous capsule (FC) surrounding the implants. (a) LGPBP and PTFE. (b) PET. The stain was hematoxylin-eosin, 2x.

**Figure 3:**
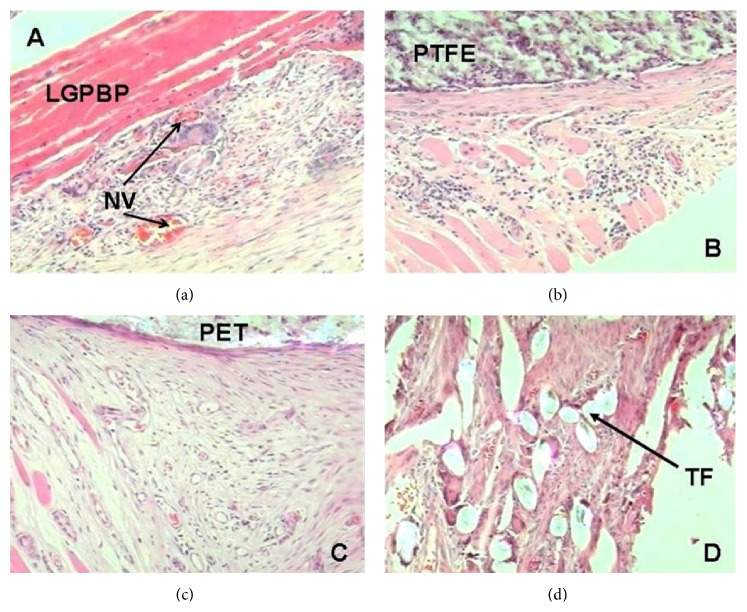
Histologic examination at the end of study of VC medialized. Showing the mild lymphoplasmacytic inflammatory infiltrate and neoformed vessels (NV) around the implants. (a) LGPBP, (b) PTFE, (c) PET, and (d) TF. The stain was hematoxylin-eosin, 10x.

Histologically, the implants of PTFE, PET, and TF were observed as amorphous fibrillar structures, whereas the pericardium showed well-organized elastic and collagen fibers (Figures 2 and 3).

Regarding the size of the fibrous capsule formed around the implants, the average size formed in the VC implanted with LGPBP (0.03117 mm ± 0.00510 mm) (Figure 2(a), Table 3) was smaller than those with PTFE (0.04461 mm ± 0.01505 mm) (Figure 2(a), Table 3), PET (0.06337 mm ± 0.01545 mm) (Figure 2(b), Table 3), and TF (0.15088 mm ± 0.03396 mm) (Table 3); however, when comparing between groups, the size of the fibrous capsules produced by TF was significant versus the other three materials (*p* = 0.001, ANOVA, Tukey) (Table 3).

None of the VC of the animals that completed the study showed neutrophil infiltration or calcification around the implants. However, TF implants that were infected and extruded showed severe neutrophil infiltration (*p* = 0.001, Kruskal-Wallis). In animals treated with LGPBP (Figure 3(a)), PTFE (Figure 3(b)), and PET (Figure 3(c)) showed mild inflammation with presence of lymphocytes, plasma cells, foreign body giant cells, and fibrosis, unlike animals implanted with TF (Figure 3(d)), in which inflammation was severe (*p* = 0.001, Kruskal-Wallis) (Table 3); in all cases, the reaction was limited to the periphery of the implant. Moreover all dogs developed a moderate amount of neoformed vessels. The VC medialized with PTFE and PET showed mild eosinophilic infiltration (*p* = 0.05, Kruskal-Wallis), and those with TF exhibited severe eosinophilic infiltration (*p* = 0.01, Kruskal-Wallis) compared to those with LGPBP (Table 3).

## 4. Discussion

In recent decades, many materials in form of pastes or strips have been used for VC medialization, but the perfect material has not yet been described because this material should be biocompatible, require minimal preparation, be easy to place, be readily available, have the same or similar biomechanical properties as the VC, be resistant to migration and resorption, and be easy to remove in case revision surgery is required [17, 18].

Studies in the literature have reported that PTFE is an effective material for medialization due to the fact that it is highly biocompatible [5–8, 19, 20], but in Mexico, the cost of the material is very expensive or unavailable, so it is necessary to seek other materials with these characteristics.

LGPBP is readily available, easily prepared, and has low-cost production in comparison with the synthetic materials. Moreover, treatment with glutaraldehyde and lyophilization improves its biochemical stability and reduces its antigenicity, providing good flexibility, easier surgical handling, easier placement, and good integration to biological tissues [9–13]. However, its efficacy and safety as an implant material for VC have not been reported. The purpose of this study was to compare the use of LGPBP, PTFE, PET, and TF as implants for VC medialization and to assess the endoscopic, macroscopic, and microscopic VC changes after medialization in an experimental canine model.

In this study we used materials in the form of strips for VC medialization because pastes exhibit increased absorption and implant migration, along with a chronic inflammatory reaction and fibrosis, favoring the formation of a mass after the injection, which predisposes to the formation of foreign body giant cell granulomas [21]. Additionally, pastes cannot be completely removed [5].

All microbiological cultures performed to pericardium samples were negative because the pH of the glutaraldehyde and the period of preservation time had an effect bactericidal, fungicidal, and virucidal, which is consistent with reports by other authors [22] who observed that the exposure of biological heart valves to 0.5% glutaraldehyde for 72 hours has a sterilizing effect.

The properties of the LGPBP implants, such as better flexibility, less memory, and ease of cutting, shaping, and sizing for easy insertion and removal from the VC, were a result of the use of fibrous pericardium (the outer layer of the pericardium) formed by laminated collagen fibers. These fibers undergo changes in the primary amines of the lysine and hydroxyproline residues and form inter- and intramolecular cross-links in their chains after being treated with glutaraldehyde [21–24], which improves their biochemical stability (allowing the elastic and collagen fibers to remain well-organized), improves their resistance, allows them to retain their shape, and increases their stiffness [9, 12, 13]. In contrast, synthetic materials tend to curl at the edges because they are formed by rigid amorphous fibers that maintain their memory [5, 6, 25].

The inflammation and edema observed with endoscopy in all VC during the first week after surgery occurred because whenever an implant is placed, an inflammatory reaction occurs in the surrounding tissue in the periphery of the prosthesis during the first hours or days after implantation and disappears over time. This is consistent with reports by de Souza et al. [3], Zhang et al. [26], and Ruijgrok et al. [27], who have studied the use of different materials as implants. Moreover, the reduced inflammatory response observed in the VC that were implanted with LGPBP occurred because this is a biologically inert material, which, unlike the synthetics, is less porous, more flexible, and has more tissue-like characteristics; therefore, it produces less foreign body reaction, as described by other authors who have used different synthetic materials for medialization [3, 5, 6]. The extrusion of the implant observed in the VC that were treated with TF was due to the synthetic material causing a severe chronic granulomatous reaction in which an abscess and fistula were formed, through which the implant was ejected [3, 21].

None macroscopic absorption was observed of LGPBP implants because the cross-links formed after treatment with glutaraldehyde make collagen able to withstand high temperatures, extreme pH, and the action of proteases. In this study, treatment with 0.5% glutaraldehyde was used because concentrations below 0.1% reduce to resistance to collagenases [13, 22–24, 27, 28]. This coincides with the findings described by other authors [5, 6], who mentioned that the use of bovine collagen for VC medialization has a lower absorption rate than other materials. However, our results do not agree with those of Lee et al. [2] and Shiotani et al. [29], who report that bovine collagen is rapidly degraded; we found well-structured collagen fibers three months after implantation. Synthetic materials were not absorbed because their microporous structure allows the growth of connective tissue without inflammation similar to that which has been observed in vascular and cardiac implants, preventing their absorption [6, 19, 20]. However, the VC that were medialized with TF in the dogs that reached the end of the study showed severe chronic inflammation due to the structure being less porous and having a rough surface.

The fibrous capsule observed around all implants is consequence of lower degree of displacement of the implant and the chronic inflammation that occurs. Nevertheless, this is considered a local repair process in which the tissue cavity that was dissected to place the prosthesis acts as a surgical wound that has to heal, and the scar tissue advances to surround the foreign body that cannot be absorbed, thus forming a fibrous capsule that can last for years [21, 30, 31]. This finding has also been observed by other authors who have described their experience with the use of various materials as implants for VC medialization [2, 3, 5, 6, 21, 29]; however, none of the authors have reported the size of the fibrous capsule. In this study, we measured the thickness of the capsule and observed no statistically significant differences regarding its size between LGPBP, PTFE, and PET implants because the three materials produced a similar chronic inflammatory reaction, unlike TF, which produced a more severe reaction.

Neither of the animals that completed the study showed neutrophil infiltration because the histological evaluation was carried out at the end of study, and these inflammatory cells usually appear during the first 24 to 48 hours following the insertion of the implant and disappear after an average of 6 weeks [21]. However, in the animals with TF that became infected and extruded, severe neutrophil infiltration was present as a result of acute inflammation that was caused by the presence of infectious agents and foreign material, and the neutrophils appeared to participate in the removal of destroyed tissues by phagocytosis and enzyme release, as well as formation of chemotactic factors [30, 31].

The inflammation with lymphocytes, plasma cells, foreign body giant cells, and fibrosis observed in all animals indicates that none of the implants were absorbed, causing a chronic granulomatous reaction, which always ends in a healing process [21]. The presence of lymphocytes occurred because these cells appeared to identify the antigen (implants) and then differentiated into plasma cells to produce antibodies. Meanwhile, foreign body giant cells were formed by the fusion of macrophages that were attempting to digest the implants. Likewise, fibrosis and neoformation of blood vessels developed because, being unable to digest the implant, fibroblasts and vascular endothelial cells surrounded it and began to proliferate, forming collagen fibers and blood vessels, to continue the wound healing process [30, 31]. The results of this study are consistent with other experimental and clinical studies that have shown that the implantation of pericardium, PTFE, and PET in various tissues does not cause a significant granulomatous reaction [5, 6, 9–13, 19, 21, 25], unlike Teflon, which causes severe reactions [21, 29].

The presence of eosinophilic infiltration in the VC after medialization with PTFE, PET, and TF may indicate that all three have allergic potential (minimal in PTFE and PET and severe in TF), perhaps because they are synthetic materials. These findings are consistent with those described by Durucu et al. who studied the medialization laryngoplasty with PTFE [6]. However, after careful review of the literature, we did not find any other report of an allergic tissue reaction with this material in the VC or in the heart and blood vessels. Additionally, there are also no reports of this reaction with the use of PET and TF in the VC, possibly because the former material was not tested in such surgical procedures and the latter has not been used in the form of strips for medialization, though the presence of eosinophilic infiltration has also not been reported with the use of these materials in cardiovascular surgery.

## 5. Conclusions

We can conclude that the LGPBP could be used as an implant for VC medialization because it is biocompatible, readily available, easy to handle and remove during surgical procedures, nonabsorbable, and nonmigrant or extrudable, requires minimal preparation, and produces a similar inflammatory reaction to that of PTFE and PET.

## Figures and Tables

**Table 1:** Differences in the surgical handling of the materials and statistical significance of the findings: (∗) statistical significance of the findings.

Surgical handling of the implants				
LGPBP	PTFE	PET	TF	*p* value^∗^
Easy to fold, cut, and give shape	Difficult to cut and give shape	Difficult to cut and give shape	Difficult to fold, cut, and give shape	
Easy to insert and remove from the microfenestra	Easy to fold, insert, and remove from the microfenestra	Easy to fold, insert, and remove from the microfenestra	Difficult to insert and remove from the microfenestra^∗^	0.005 Kruskal-Wallis
Great flexibility and less memory^∗^	Less flexibility and great memory	Less flexibility and great memory	Less flexibility and great memory	0.005 Kruskal-Wallis

Lyophilized glutaraldehyde-preserved bovine pericardium (LGPBP), polytetrafluoroethylene (PTFE), polyethylene terephthalate (PET), and Teflon felt (TF).

**Table 2:** Endoscopic changes of VC after medialization and (∗) statistical significance of the findings.

Endoscopic findings after medialization of vocal cords with different implants					
	Study groups				
	LGPBP (*n* = 18)	PTFE (*n* = 6)	PET (*n* = 6)	TF (*n* = 6)	*p* value^∗^
Motility of VC during all study time	18	6	6	6	
Volume maintenance and position of VC with respect to the glottic after medialization	18	6	6	0^∗^	0.001 Kruskal-Wallis
Degree of inflammation and edema of the VC during the first postoperative week	18 mild	6 moderate	6 moderate	6 severe^∗^	0.001 Kruskal-Wallis
Disappearance of inflammation	2 weeks after surgery	2 weeks after surgery	2 weeks after surgery	4 weeks after surgery	
Extrusion of the implant	0	0	0	3^∗^	0.005, Kruskal-Wallis

Lyophilized glutaraldehyde-preserved bovine pericardium (LGPBP), polytetrafluoroethylene (PTFE), polyethylene terephthalate (PET), and Teflon felt (TF).

**Table 3:** Microscopic thickness of fibrous capsule (mean ± standard error) formed around the implants, degree of inflammation and main inflammatory cells observed at the end of study, as well as (∗) statistical significance of the findings.

	Microscopic findings after medialization of vocal cords with different implants				
	Study groups				
	LGPBP (*n* = 18)	PTFE (*n* = 6)	PET (*n* = 6)	TF (*n* = 6)	*p* value^∗^
Thickness of fibrous capsule in mm mean ± standard error	0.03117 ± 0.00510 mm	0.04461 ± 0.01505 mm	0.06337 ± 0.01545 mm	0.15088 ± 0.03396 mm^∗^	0.001 ANOVA, Tukey
Degree of inflammation	18 mild	6 mild	6 mild	6 severe^∗^	0.001 Kruskal-Wallis
Neutrophil infiltration	0	0	0	3^∗^	0.001 Kruskal-Wallis
Eosinophilic infiltration	0^∗^	6 mild	6 mild	6 severe	0.05 Kruskal-Wallis

Lyophilized glutaraldehyde-preserved bovine pericardium (LGPBP), polytetrafluoroethylene (PTFE), polyethylene terephthalate (PET), and Teflon felt (TF).
